# Optimization of Tagged MRI for Quantification of Liver Stiffness Using Computer Simulated Data

**DOI:** 10.1371/journal.pone.0111852

**Published:** 2014-10-31

**Authors:** Serena Monti, Giuseppe Palma, Monica Ragucci, Lorenzo Mannelli, Marcello Mancini, Anna Prinster

**Affiliations:** 1 IRCCS SDN Foundation, Naples, Italy; 2 Institute of Biostructure and Bioimaging, National Research Council, Naples, Italy; 3 Radiology Department, Memorial Sloan-Kettering Cancer Center, New York, New York, United States of America; University of Pisa, Italy

## Abstract

The heartbeat has been proposed as an intrinsic source of motion that can be used in combination with tagged Magnetic Resonance Imaging (MRI) to measure displacements induced in the liver as an index of liver stiffness. Optimizing a tagged MRI acquisition protocol in terms of sensitivity to these displacements, which are in the order of pixel size, is necessary to develop the method as a quantification tool for staging fibrosis. We reproduced a study of cardiac-induced strain in the liver at 3T and simulated tagged MR images with different grid tag patterns to evaluate the performance of the Harmonic Phase (HARP) image analysis method and its dependence on the parameters of tag spacing and grid angle. The Partial Volume Effect (PVE), T1 relaxation, and different levels of noise were taken into account. Four displacement fields of increasing intensity were created and applied to the tagged MR images of the liver. These fields simulated the deformation at different liver stiffnesses. An Error Index (EI) was calculated to evaluate the estimation accuracy for various parameter values. In the absence of noise, the estimation accuracy of the displacement fields increased as tag spacings decreased. EIs for each of the four displacement fields were lower at 0° and the local minima of the EI were found to correspond to multiples of pixel size. The accuracy of the estimation decreased for increasing levels of added noise; as the level increased, the improved estimation caused by decreasing the tag spacing tended to zero. The optimal tag spacing turned out to be a compromise between the smallest tag period that is a multiple of the pixel size and is achievable in a real acquisition and the tag spacing that guarantees an accurate liver displacement measure in the presence of realistic levels of noise.

## Introduction

The increasing interest in the development of a non-invasive tool to assess liver fibrosis had led to several studies on Magnetic Resonance Elastography (MRE) [1], [2]. MRE images the propagation of shear waves, generated by an external device, whose transmission speed depends on liver stiffness. MRE has shown a high correlation between liver mechanical properties and fibrosis stages [3]–[7], but it requires a special-purpose external vibration source as well as dedicated acquisition sequences. Furthermore, it allows the evaluation of the right hepatic lobe only.

The use of the heartbeat as a transient motion source that deforms the liver during the cardiac cycle has been proposed as a more practical alternative to induce motion and deformation in the left lobe of the liver. The left lobe is a region approached with difficulty by other methods such as elastosonography and MRE because of the local anatomy and cardiac pulsatility artifacts [8]. To measure the deformations, which are supposed to be related to liver stiffness and therefore could be used as an index of fibrosis, investigators have proposed a method based on the use of tagged Magnetic Resonance Imaging (MRI). In the last 20 years, tagged MRI has been used for the non-invasive measurement of material displacements and deformations in normal and diseased hearts [9], [10]; when thin planes perpendicular to an imaging slice are selectively saturated prior to data acquisition, dark stripes or grid patterns appear on the resulting image. The tagged grid persists for a duration in the order of the longitudinal relaxation time T1, thus remaining visible for a significant fraction of the cardiac cycle. If tagging is incorporated at the beginning of a cine sequence, the change in the shape of the saturation pattern on the image during the cardiac cycle reflects the local motion of the underlying tissue. Deformation and strain can then be measured through direct estimation (analysis of the Harmonic Phase (HARP) images [11], [12]) or indirect estimation (tissue point tracking [13]) of the displacement field. The use of tagged MRI to quantify cardiac-induced strain in the liver has shown different patterns of liver motion and deformation in cirrhotic patients and healthy subjects [8], [14], as well as in two cirrhotic patient groups stratified by Child-Pugh scores [15]. Liver displacements induced by cardiac pressure range from less than 1 mm in cirrhotic patients to 5 mm for normal liver and involve a very limited part of the liver close to the bottom wall of the heart, identifiable with the second segment of the liver. It is thus very important to define an acquisition protocol that identifies the optimal angle and tag spacing of the grid applied to the images to maximize the sensitivity to these small liver displacements (that are in the order of a typical pixel size).

Computer simulations [16], [17] are particularly useful to assess the effect of varying imaging parameters [18], [19] and to validate tag quantification methods: the deformation is exactly known, non-ideal equipment behavior is not present to confound data interpretation, deformations can be modeled that are not easily generated with real phantoms, and a large number of tests can be done without incurring the cost of MRI system usage. Previous studies have focused on the presentation of computer simulation methods. Crum et al. [17] developed a software to reproduce tagged images in the frequency domain, although they were dependent on the simulated acquisition sequence. In [16], the authors added noise to their simulation and explained how deformations could be included in the model, considering only deformations induced by radially varying contraction and reproducing the heart movements. Other studies assessed the effect of varying imaging parameters. Atalar et al. [18] developed a mathematical model to optimize the tag thickness for tissue point tracking analysis methods, while Reeder et al. [19], using Bloch equation simulations, studied the tag contrast in different acquisition sequences.

The aim of this paper is to use a computer simulation study designed to reproduce a quantification model of cardiac-induced strain in the liver using tagged MRI. Additionally, it aims to evaluate the performance of the HARP image analysis method and its dependence on fine-tuning of the tag spacing and grid angle parameters that are currently selected in a heuristic way. An optimized clinical acquisition protocol for liver stiffness assessment with tagged MRI was proposed to maximize the technique's sensitivity and to allow detectability of smaller variations of the mechanical properties of the liver.

## Materials and Methods

The simulation study was completely performed using a library developed in-house for MATLAB (MATLAB R2013a, MathWorks, Natick, MA). The program generated several sets of tagged MRI images of the liver with various acquisition parameters and known deformations, which simulated liver movements during the cardiac contraction.

### Data generation

To simulate realistic tagged MRI images with variable acquisition parameters, digital grids were generated as the product of two orthogonal sets of periodic patterns of tags. Two different grid orientations (0 and 45 degrees) were combined with tag spacings varying from 4 to 7 pixels and a tag thickness equal to the nearest integer value of a third of the tag spacing. The simulated two-dimensional images were obtained by multiplying each simulated grid by a noise-free liver MRI image of 320×320 pixels.

To take into account the Partial Volume Effect (PVE), which occurs in real images when the spacing between lines of the grid is not a multiple of the pixel size, we used a step size of 0.1 for tag spacing variation. Corresponding images were accordingly generated by tagging the interpolated original image (an oversampling factor of 10 was applied in each spatial direction) with a proper grid. The actual 320×320 matrix was then reconstructed using an integral subsampling of the tagged image.

**Figure 1:**
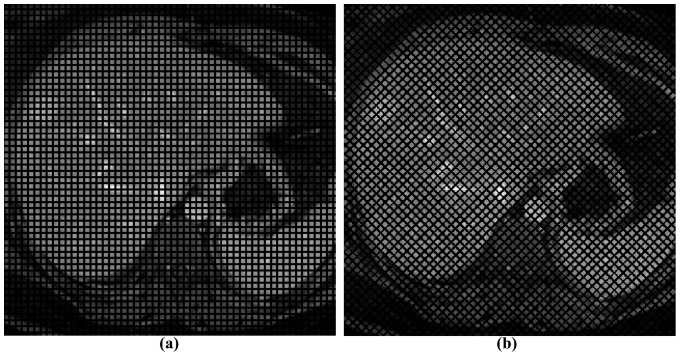
Undeformed images. Images with a tag spacing of 6 pixels and grid angle of (a) 0° and (b) 45°.

In this way, we obtained a total of 62 different simulated undeformed images (Figure 1).

### Object deformation

**Figure 2:**
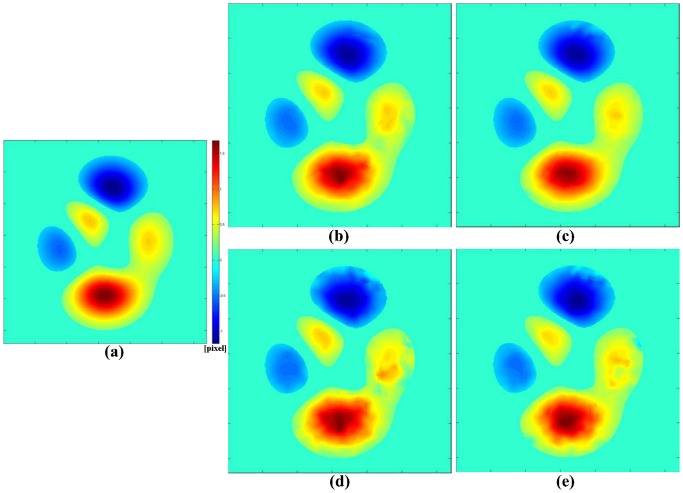
Ground truth and computed displacement maps. (a) Map of the deformation field Dg_2_ applied in both the x and y directions (Dg_2x_  =  Dg_2y_  =  [−1.2, 1.7] pixel) of the undeformed image. (b) Estimated displacement map in the x direction (Dc_x_) and (c) in the y direction (Dc_y_), obtained from tagged MRI images with grid angle of 0°, tag spacing of 5 pixels, and deformed by Dg_2_. (d) Estimated displacement maps in the x direction (Dc_x_) and (e) in the y direction (Dc_y_) obtained from tagged MRI images with a grid angle of 0°, tag spacing of 5 pixels, and deformed by Dg_2_ in the presence of a noise level of 3.5%.

To obtain the deformed images, four 2D continuous displacements maps (Figure 2a) of increasing amplitude were created by generating two smooth and well behaved functions for each amplitude using MATLAB's “peaks” function to describe the horizontal and vertical displacements, respectively (ground truth Dg_1x_  =  Dg_1y_  =  [−0.4, 0.6] pixels; Dg_2x_  =  Dg_2y_  =  [−1.2, 1.7] pixels; Dg_3x_  =  Dg_3y_  =  [−2.0, 2.8] pixels; Dg_4x_  =  Dg_4y_  =  [−2.7, 3.9] pixels). For each displacement map, the corresponding image was generated by interpolating the original image on the deformed grids.

As in real tagged MRI, tags persisted according to the longitudinal relaxation time of the tissue. The T1 relaxation effect was taken into account, simulating tag fading in the deformed images within frames during the cardiac cycle. This fading was modelled by multiplying the magnetization of the tags by the factor 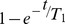, where *T_1_* = 850 ms is the estimated longitudinal relaxation time of the liver at 3T [20] and *t* = 400 ms is the time frame considered to simulate the end-systolic cardiac phase in the worst case of a very low heart rate.

**Figure 3:**
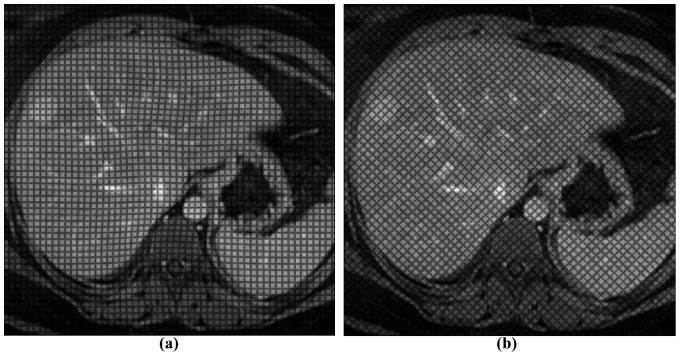
Deformed images. Images with tag spacing of 6 pixels and grid angle of (a) 0° and (b) 45°, deformed according to Dg_4_. Fading of the grid is also applied.

In summary, for each undeformed image, we obtained four images deformed by displacement maps of different amplitude that simulated the end-systolic frame for different values of liver stiffness (Figure 3).

### Noise modeling

To include noise in the simulation, the simulated images were corrupted with different levels of Rician noise [21]. In this model, the real and imaginary parts of the complex MRI images are considered to be corrupted by white additive Gaussian noise with the same noise variance that is transformed into Rician noise by taking the magnitude of the complex data:
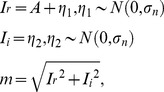
(1)where 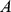 is the noise free image, 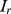 is the real component, 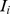 is the imaginary component, 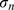 is the standard deviation of the added white Gaussian noise 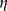, and 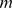 is the noisy magnitude image. To determine a realistic power of noise for our simulations, we measured the variance of a background region in a real acquired image, where the noise has a Rayleigh distribution. The noise power 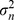 was estimated from the measured variance 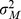 using the relation described in [22]:



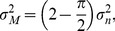
(2)As the value of 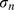 obtained in real tagged images acquired at 3T was 2.5% of the maximum intensity of the image, for our simulation, we tested realistic SNRs of 1.5, 2.5, and 3.5%. In summary, for each noise level, 31 different tag spacings at two different orientations were considered for the four deformation fields for a total of 248 simulations.

### Data analysis

The displacement maps for each simulation were obtained with in-house software that implements a HARP-based algorithm [11], [12] improved by a Gabor filter bank [23].

A tagged image can be considered to be composed of two spatial signals: the background anatomy, located at low frequencies in the Fourier domain, and the overlapped grid, whose signal is represented by two sets of orthogonal harmonic spectral peaks (one for each periodical pattern of tags) centered at multiples of the tagging frequency. The idea of the HARP algorithm is that the spectral energy corresponding to the motion of the tissue is localized around the first harmonic spectral peak of each set.

To extract this motion information, the groups of images (one undeformed and four deformed) with the same grid parameters were input into a Gabor filter bank. The filter bank was centered at a spatial frequency that corresponded to the first harmonic spectral peak of a periodical pattern of tags. The operation was repeated while changing the central frequency of the bank to extract the peaks corresponding to the other direction of the tags.

In summary, for each periodical pattern of tags, we chose a bank composed of nine filters centered around 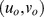, the frequency of the first harmonic peak in the Fourier domain, which can be expressed in polar coordinates as:
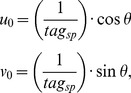
(3)with 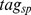 equal to the tag spacing (measured in pixels) and 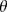 equal to the orientation of the set of lines considered. The exact position in the Fourier domain of each filter is 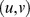:
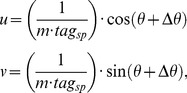
(4)where 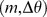 belongs to the Cartesian product of 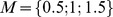 and 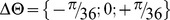 to cover typical tag rotations and translations.

For both tag directions, the final filter output was calculated by a voxel-based interpolation among the three strongest filter responses at each point in the image, obtaining two complex images, which are the result of an inverse Fourier transform of an asymmetric spectrum and are called harmonic images:
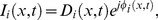
(5)where 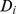 and 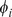 are the magnitude and phase images, respectively, and *i* indicates the spectral peak from which the harmonic image is derived (i = 1, 2). Defining the location of the spectral peaks as 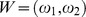 the information about the 2D displacement field of the image, 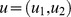, is contained in the phase image, in fact:



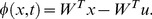
(6)From (6), the displacement field was computed by subtracting the unwrapped phase images obtained from a deformed image and the corresponding undeformed one:
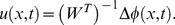
(7)


The computed displacement maps are denoted as Dc*_i_* (Figure 2).

**Figure 4:**
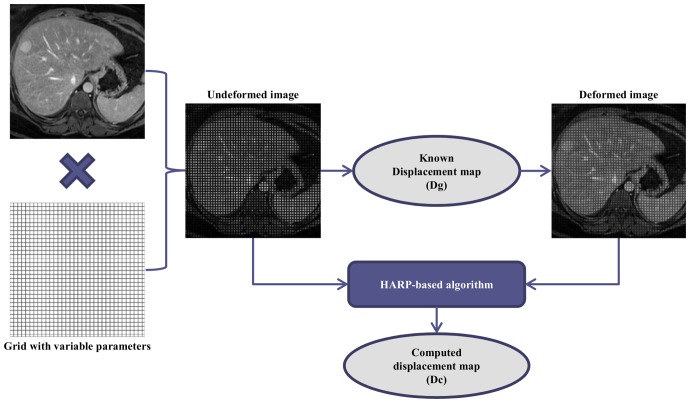
Simulation algorithm. Schematic representation of data generation and data processing steps.

The overall process of data generation and processing is schematically represented in Figure 4.

### Statistical evaluation

To evaluate the estimation accuracy for each set of grid parameters (grid orientations  = 0° and 45°, tag spacings  = 4∼7 pixels), applied displacement (Dg_ix_ and Dg_iy_, i = 1, 2, 3, 4), and three noise levels (1.5, 2.5, and 3.5%), an Error Index (EI) was calculated as:
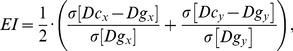
(8)where *Dc_x_*, *Dg_x_*, *Dc_y_*, and *Dg_y_*, respectively represent the estimated and applied displacement maps in the x and y directions, and 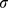 represents the standard deviation operator over the voxel sample. We calculated EI for all simulations to evaluate how the amplitude of the applied deformation, tag spacing, grid angle, and noise level influence the estimation accuracy.

A pair-sample one tailed t-test was used to determine the statistical significance of differences in the EI trends. Statistical analysis was performed using R (R Foundation for Statistical Computing, Vienna, Austria (http://www.R-project.org). A threshold of p<0.05 was considered to be statistically significant.

## Results

**Figure 5:**
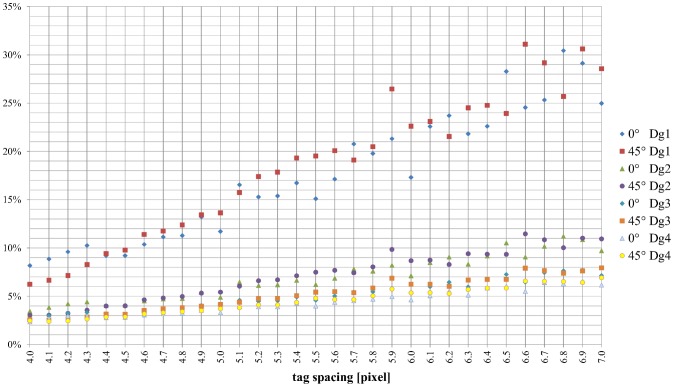
EI in a noise-free simulation. Plot of EI for each Dg_i_ and for grid angles of 0° and 45° as a function of tag spacing.

EI values were plotted for each ground truth and both grid angles as a function of tag spacing for the noise-free images (Figure 5). Data were fitted with a linear function, as shown in Table 1, where slope (m_Dgi, angle_) and intercept (q_Dgi, angle_) values for each Dg_i_ as well as both 0° and 45° grid angles were reported. In the absence of noise and for each ground truth, the estimation accuracy of the displacement field increased as the tag spacing decreased (all regression slopes were positive) over the observed tag spacing interval.

**Table 1:** Error index in noise-free simulation fitted to linear equation y =  mx +q.

*Displacement map*	*0°*			*45°*		
	*m*	*Q*	*R^2^*	*M*	*q*	*R^2^*
*Dg_1_*	7.1·10^−2^	−2.1·10^−1^	0.93	8.0·10^−2^	−2.6·10^−1^	0.95
*Dg_2_*	2.5·10^−2^	−6.7·10^−2^	0.94	2.8·10^−2^	−8.4·10^−2^	0.96
*Dg_3_*	1.7·10^−2^	−4.2·10^−2^	0.96	1.9·10^−2^	−5.2·10^−2^	0.97
*Dg_4_*	1.4·10^−2^	−3.2·10^−2^	0.96	1.5·10^−2^	−3.9·10^−2^	0.98

For all tag spacings and both grid orientations, the EIs were smaller when the amplitude of the ground truth was higher. Furthermore, the differences in EI values obtained for the various tag spacings decreased for increasing Dg. In particular, for the two grid orientations, both the absolute value of the intercept and the slope of the regressions decreased with increasing Dg For displacements on the order of pixel size or less (Dg_1_), the EIs dropped from a value of 30% for a tag spacing of 7 pixels to less than 10% for a tag spacing of 4 pixels. For greater displacements, in the order of 3–4 pixels, accuracy was also high, with higher values of tag spacing (the EI ranged between 3 and 7%).

For each ground truth, the estimation of the displacement map obtained at 0° was more accurate than the estimation at 45°, as expected because of PVE. The difference was statistically significant for Dg_1–3–4_ and approached significance for Dg_2_ (pair-sample one tailed t-test: p = 0.03 for Dg_1_, p = 0.07 for Dg_2_, p = 0.03 for Dg_3_, and p = 0.003 for Dg_4_). Moreover, for a 0° grid orientation, local minima were found to correspond to multiples of pixel size, i.e., in the absence of PVE.

Given these observations, further analyses were conducted only for a grid orientation of 0°.

**Figure 6:**
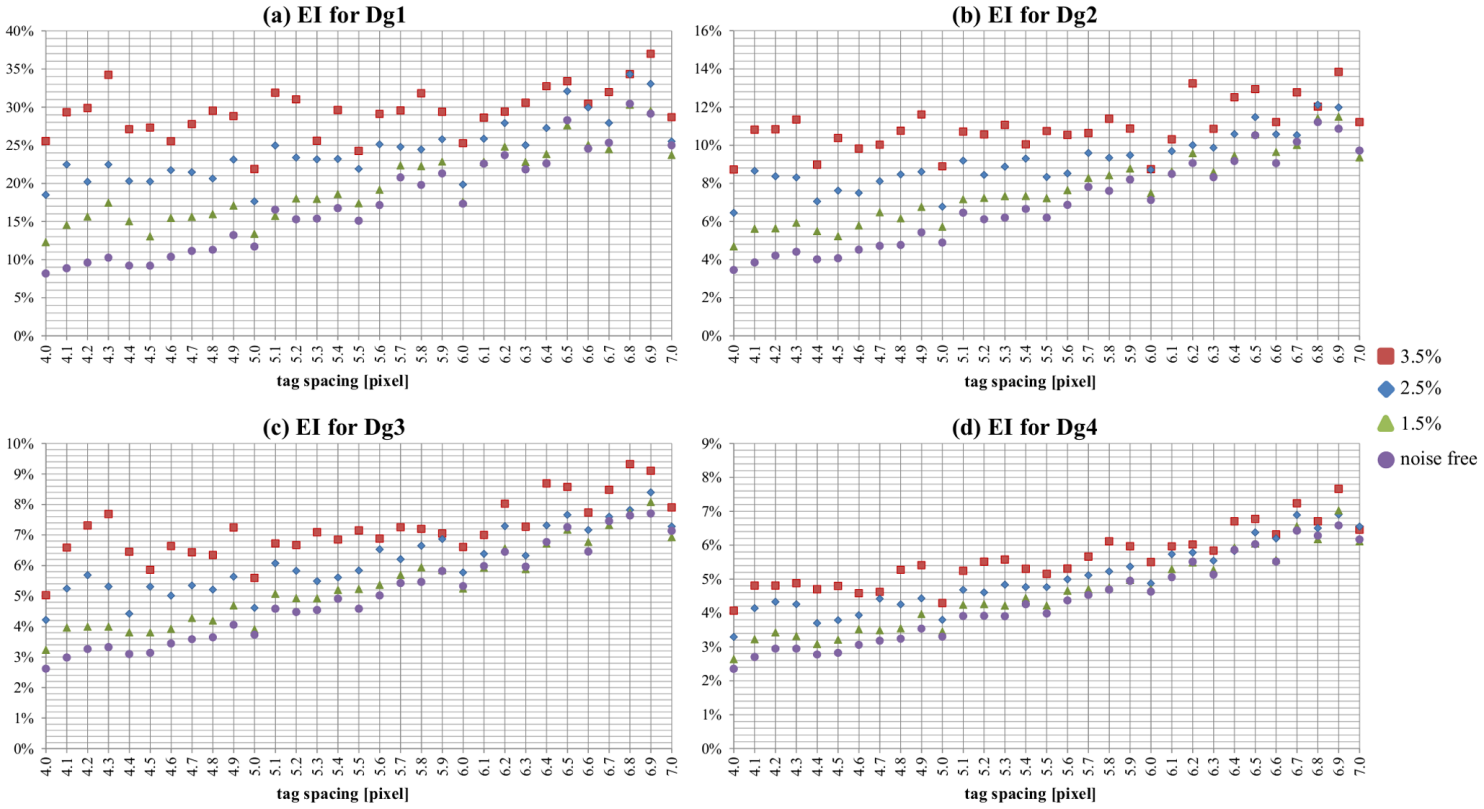
EI in the presence of noise. Plot of EI for tag spacings varying from 4.0 to 7.0 pixels, a grid angle of 0°, and different noise levels for (a) Dg_1_, (b) Dg_2_, (c) Dg_3_, and (d) Dg_4_.

When noise was added, the smoothness of the displacement measurement was affected (Figures 2(d)–(e)). Plots of EIs calculated for each Dg_i_ and the three levels of noise as a function of tag spacing are shown in Figure 6. Fitting the new calculated EIs with linear functions (Table 2), it can be observed that the slope of the linear fit decreased for each increase in the level of noise, negating almost completely the advantage of using the smallest tag spacing. This was particularly true for Dg_1_ at the maximum level of noise (the EI remained over 25%, even at tag spacing of 4).

**Table 2:** Error index at 0° in presence of noise fitted to linear equation y =  mx +q.

*Displacement map*	*1.5% noise level*			*2.5% noise level*			*3.5% noise level*		
	*m*	*q*	*R^2^*	*m*	*q*	*R^2^*	*m*	*q*	*R^2^*
*Dg_1_*	4.9·10^−2^	−7.1·10^−2^	0.82	3.6·10^−2^	4.5·10^−2^	0.64	1.5·10^−2^	2.1·10^−1^	0.19
*Dg_2_*	1.9·10^−2^	−2.9·10^−2^	0.90	1.3·10^−2^	1.9·10^−2^	0.72	8.3·10^−3^	6.3·10^−2^	0.36
*Dg_3_*	1.4·10^−2^	−2.3·10^−2^	0.92	1.1·10^−2^	2.3·10^−3^	0.84	8.0·10^−3^	2.8·10^−2^	0.56
*Dg_4_*	1.2·10^−2^	2.2·10^−2^	0.94	1.0·10^−2^	−6.5·10^−3^	0.89	9.8·10^−3^	1.9·10^−3^	0.99

The optimal tag spacing turned out to be the smallest multiple of the pixel size (4 pixels) for almost all situations considered. The very few exceptions referred to the smallest deformation in the presence of the highest level of noise, where a value of 5 was preferable.

## Discussion

We simulated several acquisition setups and demonstrated how varying tag spacing and grid angle influences the accuracy of motion estimates. We also showed how realistic levels of noise affected the results. The PVE was also taken into account for tag spacings that were not multiples of the pixel size as well as the T1 relaxation that causes grid fading.

The amplitude of the simulated displacements, for typical pixel sizes of real abdominal acquisitions, fell within the range of the observed liver movements in normal and cirrhotic patients (1∼5 mm) [8], [14], [15].

In the absence of noise, displacement estimation accuracy improved as the grid tag spacing decreased and became optimal for small displacements (pixel size or less). Comparing the values of EI at different orientations, the estimation of the displacement fields for each Dg was more accurate for a grid angle of 0° over the total range of calculated tag spacings.

The simulation of different levels of noise, similar to those measured on real acquisitions performed at 3T, showed that the smaller tag spacings, identified as optimal in the noise-free simulation, were more sensitive to the presence of noise. This is a crucial point because it is difficult to design an ad-hoc image denoising step. In fact, there is no simple linear relationship between the noise statistics in the displacement estimates and the noise statistics in the image, which undergoes a nonlinear transformation when the phase is computed.

The result of noise addition thus reduces or even cancels out the improvement in accuracy at small values of tag spacing, especially for the smallest ground truth. This imposes a lower theoretical limit on the width of the tag spacing that guarantees appropriate noise rejection. The optimal tag spacing was 4 pixels for almost all the situations considered except Dg_1_. However, the results showed EIs comparable to those obtained at a tag spacing of 5 pixels for increasing levels of noise. The lower tag spacing limit had to be combined with the physical limit of the tag spacing boundary values obtainable in a real acquisition that depends on the implementation of the tagging sequence RF pulses on the specific MR scanner used. Another parameter that is dependent on the sequence pulse implementation is tag thickness. In our simulation, we used a fixed value equal to the nearest integer to one third of the tag spacing. However, this should not be a crucial point in our analysis because the HARP method should not be substantially sensitive to variation in this parameter, unlike line tracking methods that require an accurate estimation of the tag centerline position [18].

We limited our simulation to values of T1 relaxation time that are typical for liver at 3T, but this is not an obstacle to a broader application of our results. The shorter T1 at lower magnetic field intensities [20] causes a faster tag fading and affects the CNR of the tags [24]. Hence, if we simulated an acquisition in this condition, we would expect estimation results comparable to the ones obtained with the same T1 and a higher power of noise. Analogously, the heart rate may influence the CNR of the tags, according to the Bloch equation for the T1 recovery of the tagged tissues. This is because the end-systolic cardiac phase may occur at different delays, thus affecting grid fading.

In conclusion, we suggest the use of a grid angle of 0° and, for all noise levels, a value of tag spacing that is a multiple of the pixel spacing to avoid PVE and for which the EI reached local minimums. This is because in the presence of PVE, if a pixel in a harmonic image is the mean of several complex numbers, expressed as (5), there will be an error in the displacement measurement. This occurs because the resulting phase of the pixel is not equal to the average of all the component phases, as it is a nonlinear function. Hence, the optimal tag spacing should be a compromise between the smallest tag period (a multiple of the pixel spacing) achievable in a real acquisition and the tag spacing that guarantees appropriate noise rejection in the presence of a realistic level of noise (given the scanner used for acquisition).

We believe that future work is needed to verify the impact of the optimization of the tagging MR acquisition parameters in clinical studies as well as to accurately quantify the extent of liver displacement expected at the various fibrosis stages.
